# ‘Genome skimming’ with the MinION hand-held sequencer identifies CITES-listed shark species in India’s exports market

**DOI:** 10.1038/s41598-019-40940-9

**Published:** 2019-03-14

**Authors:** Shaili Johri, Jitesh Solanki, Vito Adrian Cantu, Sam R. Fellows, Robert A. Edwards, Isabel Moreno, Asit Vyas, Elizabeth A. Dinsdale

**Affiliations:** 1 Department of Biology, 5500 Campanile Dr., San Diego State University, San Diego, CA 92128 USA; 2 College of Fisheries Science, Rajendra Bhuvan Road, Junagadh Agricultural University, Veraval, Gujarat 362266 India; 3 Computational Sciences Research Center, 5500 Campanile Drive, San Diego State University, San Diego, CA 92128 USA

## Abstract

Chondrichthyes - sharks, rays, skates, and chimeras, are among the most threatened and data deficient vertebrate species. Global demand for shark and ray derived products, drives unregulated and exploitative fishing practices, which are in turn facilitated by the lack of ecological data required for effective conservation of these species. Here, we describe a Next Generation Sequencing method (using the MinION, a hand-held portable sequencing device from Oxford Nanopore Technologies), and analyses pipeline for molecular ecological studies in Chondrichthyes. Using this method, the complete mitochondrial genome and nuclear intergenic and protein-coding sequences were obtained by direct sequencing of genomic DNA obtained from shark fin tissue. Recovered loci include mitochondrial barcode sequences- Cytochrome oxidase I, NADH2, 16S rRNA and 12S rRNA- and nuclear genetic loci such as 5.8S rRNA, Internal Transcribed Spacer 2, and 28S rRNA regions, which are commonly used for taxonomic identification. Other loci recovered were the nuclear protein-coding genes for antithrombin or SerpinC, Immunoglobulin lambda light chain, Preprogehrelin, selenium binding protein 1(SBP1), Interleukin-1 beta (IL-1β) and Recombination-Activating Gene 1 (RAG1). The median coverage across all genetic loci was 20x and sequence accuracy was ≥99.8% compared to reference sequences. Analyses of the nuclear ITS2 region and the mitochondrial protein-encoding loci allowed accurate taxonomic identification of the shark specimen as *Carcharhinus falciformis*, a CITES Appendix II species. MinION sequencing provided 1,152,211 bp of new shark genome, increasing the number of sequenced shark genomes to five. Phylogenetic analyses using both mitochondrial and nuclear loci provided evidence that *Prionace glauca* is nested within *Carcharhinus*, suggesting the need for taxonomic reassignment of *P*. *glauca*. We increased genomic information about a shark species for ecological and population genetic studies, enabled accurate identification of the shark tissue for biodiversity indexing and resolved phylogenetic relationships among multiple taxa. The method was independent of amplification bias, and adaptable for field assessments of other Chondrichthyes and wildlife species in the future.

## Introduction

Chondrichthyes (sharks, rays, skates, and chimaeras) are among the oldest extant vertebrates. They are vital to top-down regulation and maintenance of oceanic ecosystems, and to sustain healthy commercial fisheries and ecosystem services derived from marine environments. The high demand for shark and ray fins and an emerging demand for shark meat and oil^1^ has made world trade in Chondrichthyes a >$US 800 million industry^1^. Demand for shark fins and meat has caused overfishing and exploitation of Chondrichthyes worldwide, driving an unprecedented number of chondrichthyan species towards extinction^2^. Of the ~1,178 chondrichthyan species known today, 25% are threatened with extinction globally^2^, making Chondrichthyes one of the most endangered vertebrate species groups. The threat is even more severe in areas such as the Arabian Seas Region (ASR) with up to 50% of chondrichthyan species in the region facing extinction threat^3^.

Chondrichthyan fisheries are driven by 20 nations^2,4^ and a vast majority of these countries experience the highest levels of data deficiency^2^. An estimated 46% of Chondrichthyes are considered data deficient, suggesting that information is insufficient to determine their conservation status^2^. These data deficiencies restrict design and implementation of efficient conservation measures^2,4^ and allow continued exploitation of the most vulnerable Chondrichthyes. Not surprisingly, nations with high data deficiency also have some of the highest percentages of threatened Chondrichthyes^4^. For effective conservation of remaining chondrichthyan populations continued monitoring and assessment is essential in order to determine taxonomic units, population structure, population size and identify priority conservation units with respect to evolutionary and management significance. Once these data are available, they can be used to design effective conservation measures including but not limited to, the designation of protected areas, reduction of targeted and incidental fishing of priority concern units, and CITES listing of priority concern units to protect vulnerable populations.

Data deficiency of Chondrichthyes can be addressed by increasing the rate of ecological investigations in areas with a high density of threatened and data deficient species. In the current scenario, molecular ecological studies in Chondrichthyes are typically restricted to DNA barcoding and microsatellite analyses for taxonomic identification and population studies respectively^5^. Molecular taxonomy is conducted by PCR amplification and sequencing of DNA barcodes such as Cytochrome oxidase 1(COI)^6**–**8^ NADH2^9,10^ 16S rRNA and 12S rRNA^11,12^, of the mitochondrial genome and the Recombination-Activating Gene 1(RAG1)^13^ and Internal transcribed spacer 2 (ITS)^14^ regions of the nuclear genome. Sampling each one of these barcodes from a specimen requires *a priori* sequence information, PCR optimization, PCR amplification, post PCR clean up and finally sequencing. Single barcode PCR based identification is not the most efficient method for taxonomic identification and can be limiting due to the following reasons. First, single barcodes do not always guarantee accurate taxonomic identification, due to non-amplification for certain species using universal primers^7^, or identical barcode sequences for multiple species such as Carcharhinid sharks^15–17^ or different barcode sequences for geographically isolated populations of the same nominal species^18,19^. Second, the laboratory required for amplification and sequencing of barcodes from chondrichthyan specimens is often inaccessible to researchers and fisheries scientists in areas with heavy chondrichthyan fishing and exports, requiring them to outsource detection, adding significantly to the processing time^20^. Last, single barcode PCRs provides up to 1000 bp of data and restrict the amount of genetic data derived for the respective species. Microsatellites used for population studies among Chondrichthyes are also PCR based^5^, only include neutral genetic loci, require standardization for use across different laboratories and do not provide genome-wide assessments of neutral as well as adaptive loci. Accurate estimates of population size and structure as well as understanding of evolutionary units are only obtained from screening multiple genomic loci such as single nucleotide polymorphisms (SNPs) from genomic data sets^21–24^. Thus, PCR based approaches for taxonomic and population studies are limiting to molecular ecological studies in Chondrichthyes. Adopting a genomics approach could allow assessment of multiple genomic loci without amplification bias and without the need for *a priori* sequence information. Such an approach would be ideal to increase ecological and evolutionary investigations in Chondrichthyes, similar to those of other commercial fisheries^25^. Increasing capacity for use of genome-wide data addresses taxonomic inadequacies and enables complex ecological and evolutionary questions to be answered by providing information about population size, structure, biogeography and priority conservation units. These data together will address chondrichthyan data deficiencies and assist conservation efforts.

Genomic analyses have limited adoption among chondrichthyan researchers due to complex instrumentation and expensive sequencing for whole genome shotgun sequencing or reduced representation methods such as Restriction site-Associated-DNA-Sequencing (RADseq) and Hybridization based gene capture. A crucial aim of the current study was to reduce the burden of sample processing and analyses, and to increase accuracy, speed and breadth of ecological investigations of Chondrichthyes through genome sequencing with sophisticated yet accessible technology. We explored the use of multiple mitochondrial and nuclear barcode regions which form the high copy number fraction of the genome to increase accuracy of taxonomic identification. We explored direct sequencing of genomic DNA (gDNA) to reduce sample processing times and to avoid amplification biases, thus increasing the potential for application of the assay to multi- species assessments. ‘Genome skimming’^22^ which allows deep sequencing of high copy number fractions of the genome was used to access multiple mitochondrial and nuclear barcode sequences from shark genomic DNA. Sequencing was done on the MinION portable sequencer from Oxford Nanopore Technologies (ONT) which requires library preparation and loading times of as little as thirty minutes, allows sequence acquisition as early as 2 hours into sequencing, and allows local sequencing and analyses of genomic DNA with a user-friendly software interface installed on a portable computer.

We present characterization of ‘genome skimming’ on the MinION portable sequencer for taxonomic identification and phylogenetic analyses of Chondrichthyes. We report identification of a shark fin specimen in India’s export market to *Carcharhinus falciformis*, based on the complete mitochondrial sequence and nuclear genetic regions of the specimen. In addition, we present two sequence analyses methods which can be adapted to other wildlife species and which are suitable for standalone analyses in the field or laboratory, respectively. Last we resolved the placement of *P*. *glauca* within the Carcharhinus genus and described its relationships with other taxa in the genus, with high statistical power using the entire mitochondrial genome and nuclear genes from *C*. *falciformis*. Our sequencing and analyses methods increase the available genomic information about *C*. *falciformis* and provide an accessible tool for genome-wide studies across Chondrichthyes.

## Results

### Genus level taxonomic resolution through photo identification and COI fragment analyses

**Figure 1:**
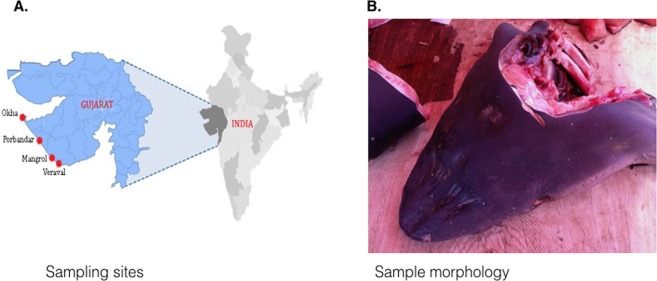
Sampling location and sample morphology of a dismembered shark specimen at time of sampling.

The shark specimen encountered in Veraval, Gujarat, was processed for international fin and meat exports, and the head and fins of the specimen could be sampled for tissue collection and photo-identification purposes (Fig. 1). Based on features of the head and mouth cavities the specimen was identified to the Carcharhinidae family. In order to obtain a species-level taxonomic resolution, we used DNA extracted from the specimen to PCR amplify three different fragments from COI and NADH2 mitochondrial loci. We were successful in amplifying one short 160 bp fragment of the cytochrome oxidase 1 (COI) gene, while PCR amplification of two longer fragments from COI and NADH2 did not yield a PCR product. The 160 bp PCR fragment was cross-referenced with the BOLD database which resulted in 100% sequence similarity to six species of the Carcharhinus genus including *Carcharhinus brevipinna*, *Carcharhinus falciformis*, *Carcharhinus melanopterus*, *Carcharhinus sorrah*, *Carcharhinus altimus* and *Carcharhinus limbatus*. These results obtained using a single short barcode supported identification based on morphology but were inconclusive for species-level identification.

### Assessment of complete mitochondrial sequence using ‘genome skimming’ on the MinION

In order to obtain higher taxonomic resolution by assessment of longer and multiple barcode sequences ‘genome skimming’ using genomic DNA of the specimen was conducted on the MinION sequencer. We obtained 74,536 sequences with read lengths between 5–98,997 bp within 36 hours of a single sequencing run.

**Figure 2:**
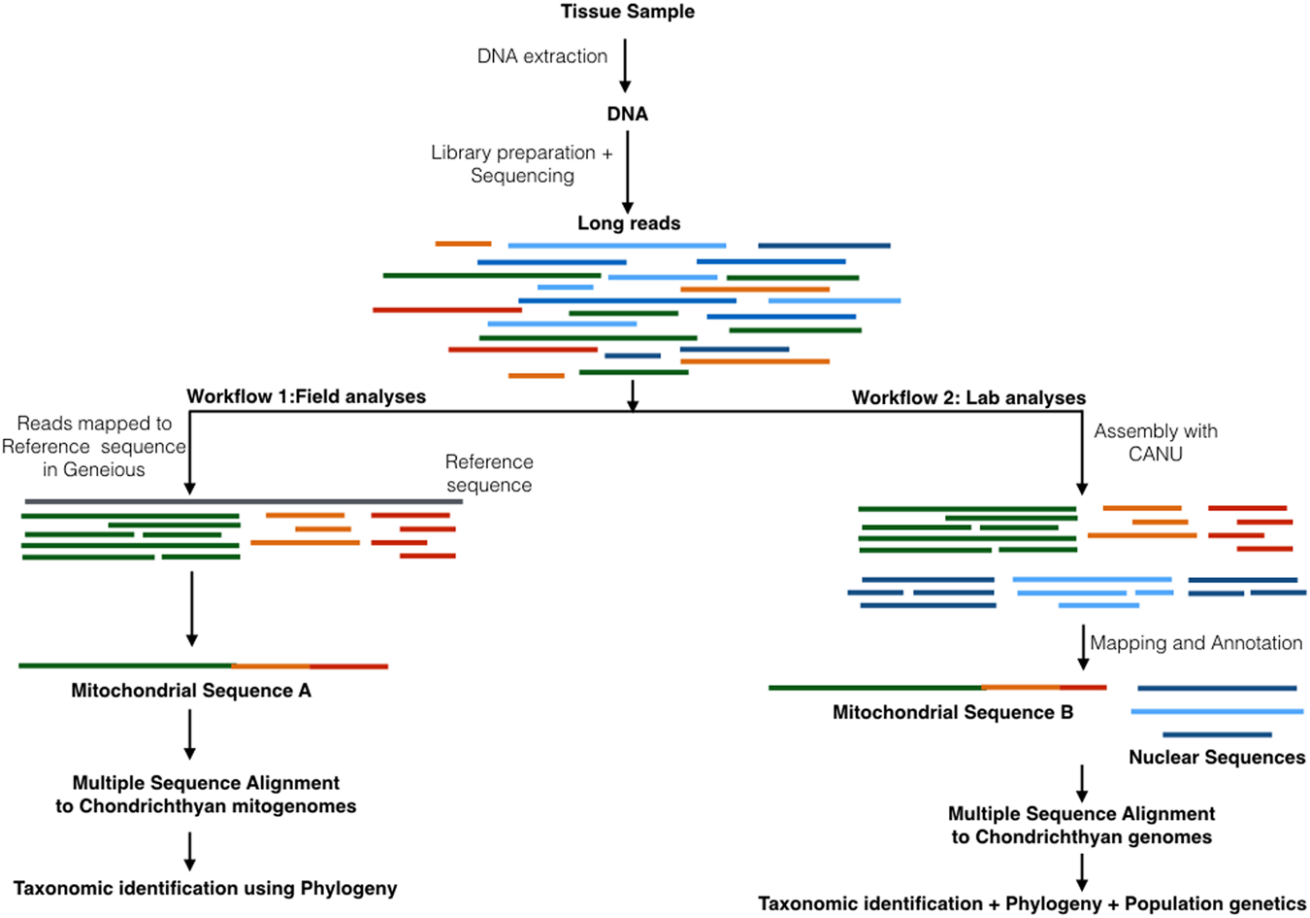
Two workflow designs used in this study one, for field or forensic applications with no connectivity and average laptop computing power and two, for detailed laboratory analyses with connectivity and high computing power.

**Figure 3:**
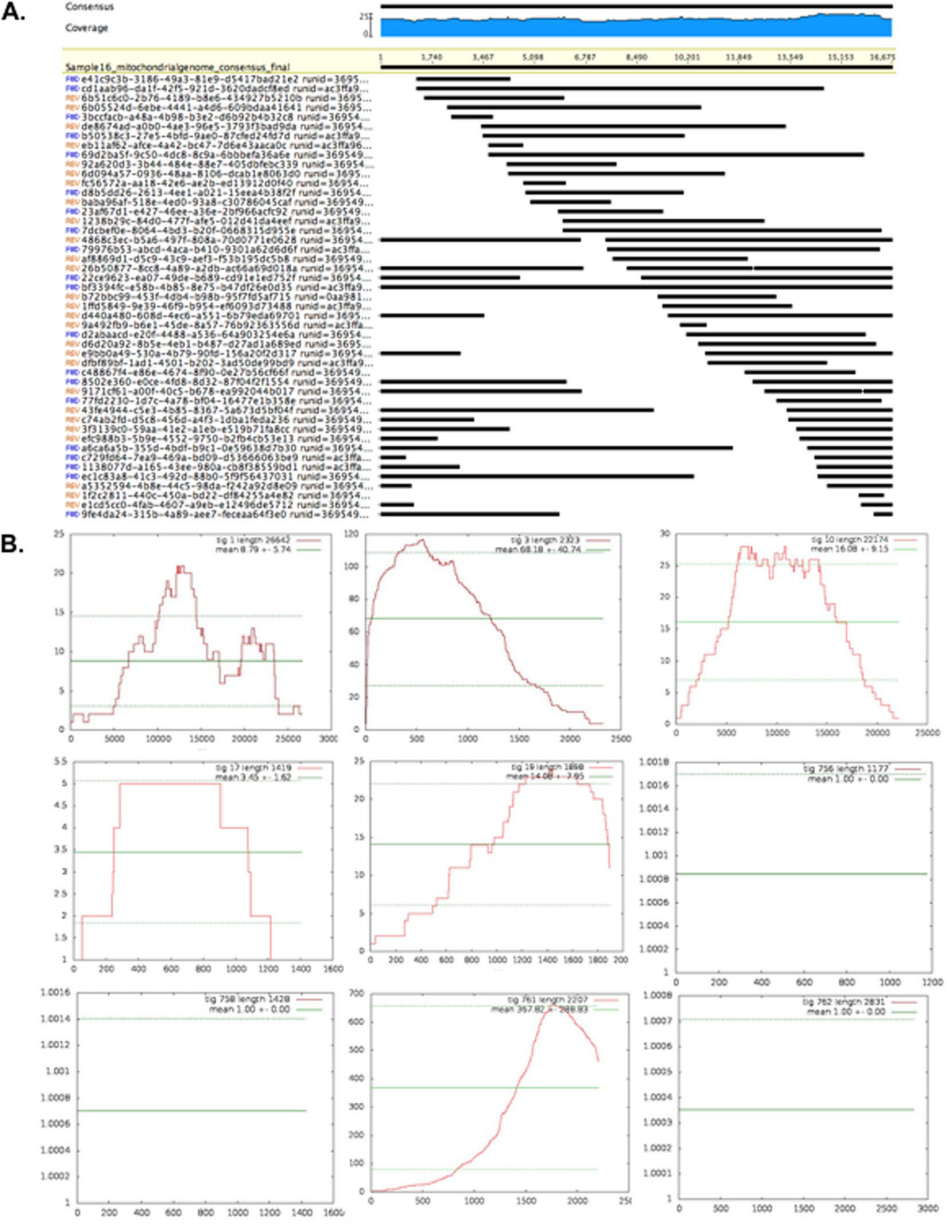
(**A**) Sequencing coverage for complete mitochondrial genome of the specimen. The mean coverage for 16, 675 bases of the mitogenome was 19.4 with a Std Dev of 2.0, and minimum and maximum coverage of 16x and 25x respectively. Coverage in the forward direction was 9.5 and 9.9 in the reverse. Sequencing coverage for regularly used individual mitochondrial barcodes is shown in Fig. S1. (**B**) Sequencing coverage for mitochondrial and nuclear genes of the specimen. Sequencing coverage for nine annotated nuclear genome contigs and one mitochondrial contig recovered with Workflow II ranged from 1x to >50x.

The sequences were analyzed using two parallel workflows (Fig. 2). In the first workflow, raw reads from the MinION were mapped to reference mitogenomes of *Carcharhinus* spp. identified to match the shark specimen by PCR fragment alignment. Mitogenomes were available for four of six *Carcharhinus* spp. (*Carcharhinus melanopterus Carcharhinus sorrah*, *Carcharhinus brevipinna* and *Carcharhinus falciformis* (Supplementary Table S1) that matched the specimen using PCR. These 4 mitogenomes were used to account for biases in the database and increase the mapping accuracy of MinION sequences. All of the 74,536 reads from the MionION were mapped onto each of the 4 mitogenomes, which identified the same set of 47 reads (~0.06%) from each mapping. Assembly of the 47 reads generated a complete mitochondrial contig of 16,675 bp (Fig. 3A, Genbank accession #MK092088), a genome size similar to mitochondrial genomes reported for *Carcharhinus* spp., (eg. *Carcharhinus sorrah* (16,707 bp), *Carcharhinus brevipinna* (16,706 bp), *Carcharhinus melanopterus* (16,706 bp), *Carcharhinus plumbeus* (16,706 bp), and *Carcharhinus obscurus* (16,706 bp)^26^, but significantly different than the published mitogenome for *Carcharhinus falciformis* at 17,639 bp^27^. Mean coverage for the complete mitochondrial genome was 19.4 × ± 2 × (Fig. 3A, Supplementary Fig. S1).

In the second workflow (Fig. 2), all 74,536 sample reads were first assembled into contigs, quality controlled by trimming read ends and aligned against a database of chondrichthyan mitochondrial and nuclear sequences to annotate the contigs. This method yielded a mitochondrial sequence of 16,477 bp which was 98% identical to the sequence in workflow 1 and nuclear genetic loci such as the Internal transcribed spacer 2 (ITS2) region, 5.8S, 28S and 18S  rRNA coding regions, and protein-coding regions for antithrombin or SerpinC, Immunoglobulin lambda light chain, Preprogehrelin, selenium binding protein 1(SBP1), Interleukin-1 beta (IL-1β) and Recombination-Activating ene 1 (RAG1) (SRA accession #PRJNA503780). With the second workflow, we obtained an almost complete mitochondrial sequence and in addition, nuclear coding and non-coding regions. Mean coverage for nuclear coding and non-coding genes recovered ranged from 1x to >50x (Fig. 3B, Supplementary Fig. S1).

**Figure 4:**
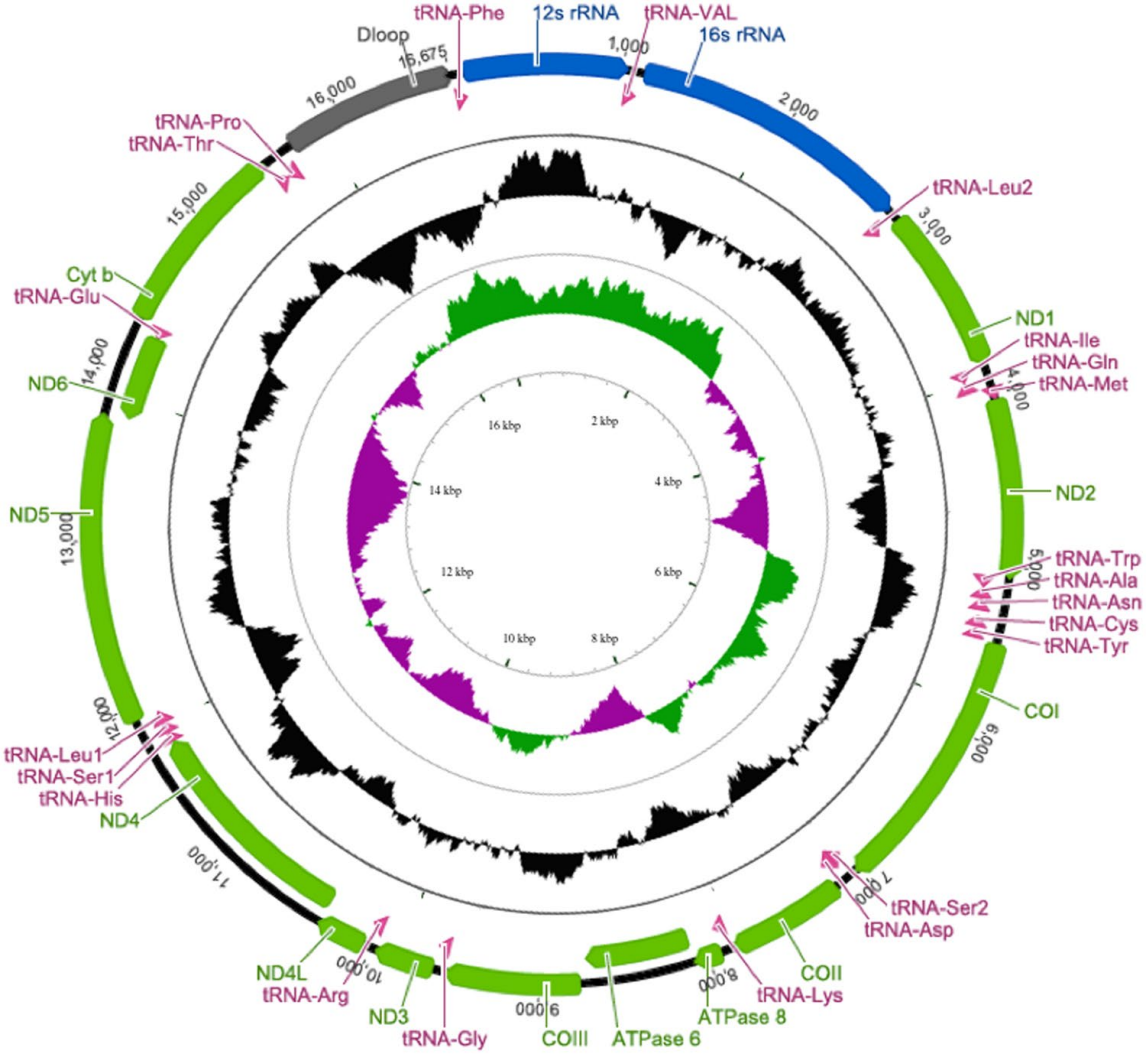
Annotated mitochondrial sequence indicating gene position and G/C richness regions. Outer circle represents aligned genes with outermost representing the heavy strand (H) and adjacent inner ring the light strand (L). Green represents protein coding regions, pink tRNA, blue rRNA, and grey is the D-loop. Middle circle in black represents GC content: Protruding outwards indicates GC rich while inward protrusion shows AT rich regions. The inner most circle is GC skew (G − C/G + C) with positive skew (between 0 and 1) represented by green and negative as purple (between 0 and −1).

Annotation of the complete mitochondrial sequence using annotation tools^28–30^ followed by manual annotation of the D-Loop region in Geneious^®^^31^ yielded recovery of all coding and non-coding regions of the mitochondrial genome including thirteen protein coding regions, two rRNA coding regions, twenty-two tRNA coding regions, a replication region and a control region (Fig. 4, Table 1). These results demonstrate the efficiency of genome skimming over conventional single barcode PCR based assays. A single sequencing run on the MinION sequencer provided the entire mitochondrial sequence equivalent of multiple mitochondrial barcodes and multiple nuclear genetic loci. These data were acquired in a very brief time frame, without any amplification bias, and with minimal infrastructure compared to PCR based studies.

**Table 1:** Genes identified in the *Carcharhinus falciformis* mitochondrial sequence with their start and end positions on the forward (+) or reverse (−) strands and a score representing the heuristic measure of the annotation content for each entry.

Start	Stop	Type	Name (Direction)	Score
1	70	tRNA	**tRNA-Phe (+)**	1.50E-14
72	1031	rRNA	**12s rRNA (+)**	0
1029	1100	tRNA	**tRNA-VAL (+)**	1.00E-14
1124	2764	rRNA	**16s rRNA (+)**	0
2764	2838	tRNA	**tRNA-Leu2 (+)**	1.40E-12
2839	3813	gene	**ND1 (+)**	453393778
3814	3883	tRNA	**tRNA-Ile (+)**	2.00E-14
3885	3956	tRNA	**tRNA-Gln (-)**	5.00E-15
3956	4024	tRNA	**tRNA-Met (+)**	6.10E-15
4025	5071	gene	**ND2 (+)**	324312000.6
5070	5140	tRNA	**tRNA-Trp (+)**	1.80E-15
5142	5210	tRNA	**tRNA-Ala (-)**	9.80E-14
5211	5281	tRNA	**tRNA-Asn (-)**	2.00E-12
5317	5384	tRNA	**tRNA-Cys (-)**	1.50E-11
5386	5454	tRNA	**tRNA-Tyr (-)**	2.60E-16
5456	7012	gene	**COI (+)**	1166241108
7013	7083	tRNA	**tRNA-Ser2 (-)**	6.40E-15
7087	7156	tRNA	**tRNA-Asp (+)**	6.30E-13
7164	7854	gene	**COII (+)**	280357893.9
7855	7925	tRNA	**tRNA-Lys (+)**	4.90E-13
7927	8094	gene	**ATPase 8 (+)**	3849114.9
8085	8719	gene	**ATPase 6 (+)**	135520179.4
8763	9548	gene	**COIII (+)**	361705805.6
9551	9619	tRNA	**tRNA-Gly (+)**	1.90E-13
9620	9970	gene	**ND3 (+)**	43327351.7
9969	10038	tRNA	**tRNA-Arg (+)**	2.90E-12
10039	10335	gene	**ND4L (+)**	25640182.3
10329	11709	gene	**ND4 (+)**	770745173.3
11710	11778	tRNA	**tRNA-His (+)**	1.40E-11
11779	11843	tRNA	**tRNA-Ser1 (+)**	5.60E-08
11844	11915	tRNA	**tRNA-Leu1 (+)**	8.30E-22
11916	13745	gene	**ND5 (+)**	1036110292
13741	14262	gene	**ND6 (-)**	47160329.7
14263	14331	tRNA	**tRNA-Glu (-)**	1.10E-11
14334	15479	gene	**Cyt b (+)**	760558631.5
15479	15550	tRNA	**tRNA-Thr (+)**	1.20E-11
15553	15620	tRNA	**tRNA-Pro (-)**	2.60E-09
15663	16674	rep-origin	**Dloop (+)**	3240692.3

### Species-level taxonomic resolution using phylogenetic analyses of multiple barcode regions

**Figure 5:**
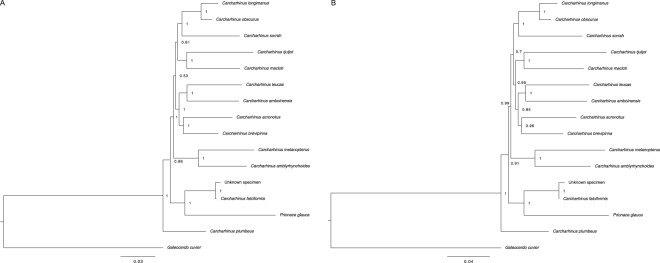
(**A**) Bayesian estimate of relationships among Carcharhinus spp. using complete mitogenomes. Bayesian phylogenetic estimate for complete mitochondrial genomes from 13 *Carcharhinus* species and *Prionace glauca*, and *Galeocerdo cuvier* as an outgroup. The unknown sample clusters with *C*. *falciformis*. Numbers at nodes are posterior probabilities. (**B**) Bayesian estimate of relationships among Carcharhinus spp. using protein coding genes. Bayesian phylogenetic estimate from 13 protein-coding mitochondrial genes from 13 *Carcharhinus* species and, *Prionace glauca*, and *Galeocerdo cuvier* as an outgroup. The unknown sample clusters with *C*. *falciformis*. Numbers at nodes are posterior probabilities.

We constructed phylogenetic trees based on the full mitochondrial genomes (Fig. 5A) and 13 protein coding regions (Fig. 5B) from the mitogenome of 13 shark species of the genus *Carcharhinus*, and *Prionace glauca*, and used *Galeocerdo cuvier* as an outgroup (family Carcharhinidae). Genbank accession numbers for reference sequences used are listed in Supplementary Tables S1 and S2. Our phylogenies based on multiple mitochondrial markers are congruent with previous phylogenetic studies in the *Carcharhinus* genus conducted using a single NADH2 locus^9,13^ albeit ours have better statistical support (Fig. 5A). Phylogenetic estimates showed robust support (>0.95 posterior probability (Fig. 5A,B), >90% bootstrapping (Supplementary Fig. S2) toward the tips of the phylogeny and lowered support at some deeper nodes. Phylogenetic estimates based on the full mitochondrial genome (Fig. 5A) showed higher support throughout than those based on protein-coding genes only (Fig. 5B). Using both analyses, based on full or protein-coding mitochondrial sequences, we recovered a sister relationship between the unknown sample and *C*. *falciformis* (Fig. 5A,B). Our analyses estimated identical phylogenetic relationships among these 15 species, including support for the placement of *P*. *glauca* within *Carcharhinus* (Fig. 5A,B).

**Figure 6:**
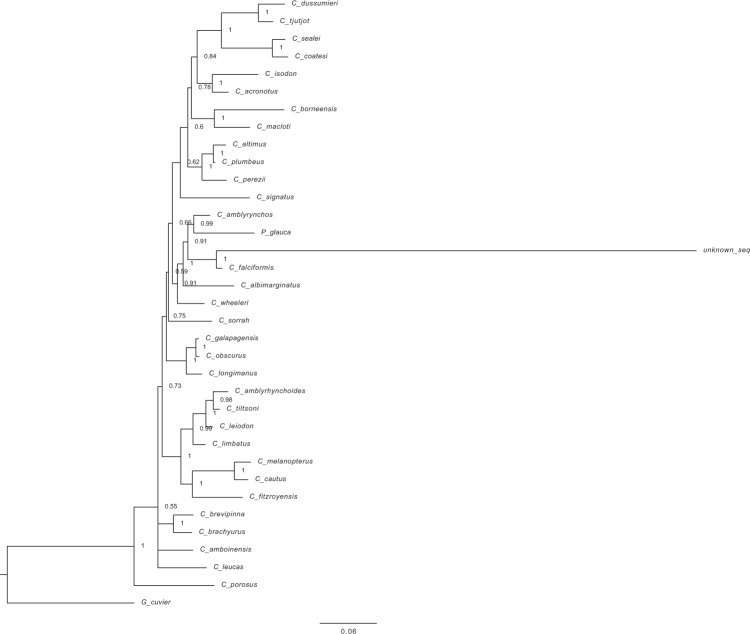
Bayesian estimate of relationships among Carcharhinus spp. using mitochondrial and nuclear genes. Bayesian phylogenetic estimate from concatenated alignment of three mitochondrial protein coding loci (NADH2, COI, NADH4) and one nuclear locus (ITS2) from 32 *Carcharhinus* species, *Prionace glauca*, and *Galeocerdo cuvier* as an outgroup. The unknown sample clusters with *C*. *falciformis*. Numbers at nodes are posterior probabilities.

We also constructed phylogenetic trees based on a concatenated sequence of three mitochondrial protein-coding sequences (NADH2, COI, NADH4) and one nuclear intergenic region (ITS2) of 33 shark species of the genus *Carcharhinus* (of 35 currently described species), with *P*. *glauca*, and *G*. *cuvier* as an outgroup (Fig. 6). Genbank accession numbers for references sequences used are listed in Supplementary Table S3. Both Maximum Likelihood and Bayesian analyses using these concatenated genes strongly supported a sister relationship between the unknown sample and *C*. *falciformis* (Fig. 6). The concatenated analyses showed robust support (>0.95 posterior probability (Fig. 6), >90% bootstrapping, (Supplementary Fig. S2) toward the tips of the phylogeny and lowered support at many deeper nodes. These results corroborate relationships seen using mitochondrial markers (Fig. 5A,B) and improve on our phylogenetic analyses of *P*. *glauca* using only mitochondrial genomes, which did not include *C*. *amblyrhynchos* due to unavailability of a complete mitogenome.

Using phylogenetic analyses based on multiple nuclear and mitochondrial regions we were able to identify an unknown shark specimen as *Carcharhinus falciformis*, a CITES Appendix II listed species.

Comparison of individual protein-coding regions of the sample sequence to *Carcharhinus falciformis* sequences in the Genbank database, demonstrated ≧99.3% accuracy of our specimen sequences (Table 2) (Genbank reference sequences used for comparison are listed in Supplementary Table S4). In addition, we improved the phylogenetic placement, improved mitochondrial sequence data and increased the amount of nuclear genomic data on this species by five-fold (1.1 million bp).

**Table 2:** Accuracy of *Carcharhinus falciformis* mitochondrial sequences based on comparison with Genbank reference sequences (Table S4).

Genetic Loci	Accuracy	Differences/Total base pairs
Cytochrome oxidase 1 (COI)	99.33–100%	0–2/600–654
NADH2	98.5%	16/1044
12s rRNA	98.5%	6–7/1357–1411
16s rRNA	98.5%	6–7/1357–1411
Mitochondria	98.75%	207/16,629–17,639*

*Only one mitochondrial reference sequence for *C*. *falciformis* with 17, 639 base pairs exists. Length of mitochondrial sequence obtained for *C*. *falciformis* in this study is 16, 629.

## Discussion

Chondrichthyes are data deficient globally^2^, making them highly vulnerable to fishing related exploitation. Molecular ecological assessments in areas of high data deficiency are often inefficient at addressing chondrichthyan deficiencies due to their reliance on molecular genetic tools that have a restrictive data output and because pan-genomic methods are inaccessible due to complex infrastructure and instrumentation requirements^2,3^. As apex predators, Chondrichthyes are important for top-down regulation of oceanic ecosystems^32^. Life history traits such as slow generation times, small litters, and stringent habitat requirements, make it challenging for chondrichthyan species populations to recover from overfishing practices^33^. Due to overexploitation from unregulated fishing, of the 1,178 chondrichthyan species, approximately 25% face extinction and ~46% are data deficient, meaning information is insufficient for species conservation and management^4^. To reduce these data deficiencies, the current manuscript describes methods that increased the taxonomic resolution of *Carcharhinus falciformis*, a Near Threatened CITES Appendix II listed species, in addition to providing genomic data for population genetic and evolutionary studies. The current study has increased the number of sequenced Chondrichthyes by 20%. The genomic data obtained, improved the genomic information for *Carcharhinus falciformis*, reduced data deficiencies through increased resolution of phylogenetic relationships within the Carcharhinus genus and in future studies will provide genomic data to understand population structure, population size and biogeography, which are important in management of priority species of concern such as the silky shark. Last, we described two different workflows for sequence analyses which will allow capacity building for taxonomic identification and population and phylogenetic studies among researchers with basic and advanced bioinformatic skills.

### Comparison of Sequencing methods

The MinION portable sequencer obtained multiple barcode sequences, along with complete mitochondrial **(**Fig. 3A, Fig. 4**)** and partial nuclear genomes (Fig. 3B) of a previously un-sequenced shark using genomic DNA collected from the market place. This method enabled accurate taxonomic identification of a shark tissue specimen by using multiple genetic barcodes, without the need for PCR amplification and without *a priori* sequence information.

We demonstrate the use of MinION sequencers to sequence larger genome sizes of 1–6.7 Gb, more typical of shark species^34–36^. Nuclear genome size in sharks varies between 1–3 Gigabases and our method achieved optimal coverage **(**Fig. 3A,B**)** of high copy number regions, such as mitochondrial and nuclear marker sequences often used for taxonomic identification and phylogenetic inference. Our methods expand on studies by Parker *et al*.^37^, who used the MinION sequencer to sequence relatively small Arabidopsis (~135 Mbp) genomes. Genome skimming using the MinION is amplification independent making it less affected by sequence biases. GC content is an important determinant of sequencing and computational bias in NGS^38,39^. The mean GC content for mitochondrial genomes of Carcharhinid sharks is 38.44% (±0.006 STDEV), similar to the mitochondria of *C*. *falciformis* at a GC content of 38.4%. The range of GC content for all 673 chondrichthyan NADH2 barcodes sequenced to date is 32–47%. The range of GC content that the MinION sequenced in the *C*. *falciformis* genome was 29–64%, suggesting a wide capability of the sequencer for genomic assessment of a variety of Chondrichthyes. The method thus has potential to aide in taxonomic identification, ecological, evolutionary and population level studies of new and understudied species. Last, although the method relies on state of the art NGS technology, it only requires gDNA extraction from the specimen which takes about three and a half hours, a 15–30 minutes library preparation/loading time and finally sequencing with the MinION and a laptop. Basecalling can be done locally on the laptop computer in parallel or post-sequencing using ONT software. Analyses for the current study did not require internet connectivity as local databases were curated in advance onto the Geneious software on laptop computers^40^. In the future, this method is adaptable to field-based ecological studies and will facilitate capacity building among conservation managers, wildlife researchers and forensics officials.

The higher cost of MinION flowcells compared to PCR amplification and sequencing of single barcodes remains a limiting factor to the wide adoption of this technology. Barcode sequencing using PCR requires gDNA extraction (~3.5 hrs), followed by PCR optimization for the species under consideration (hours to days), PCR amplification of a single barcode and possibly multiple species (~2 hrs) and last, PCR clean up (~30 minutes) and Sanger sequencing (~24 hrs) turnaround time. For the suggested timeline, the location should have access to a Sanger sequencer, which in case of the sampling site for the current study was a 7 hour drive away (403 km), suggesting that a 24 hr turnaround time is most unlikely. At the end of the PCR process a single barcode is sequenced for multiple species. In comparison, genome skimming on a MinION sequencer requires gDNA extraction (~3.5 hrs), followed by library preparation and flowcell loading (~15–30 minutes), followed by sequencing (~8–36 hours), and recovery of fastq sequences and BLAST alignment to a local or global database for taxonomic identification. Therefore, PCR-based bar coded derives ~ 150–500 bp compared with 1,152,211 bp of data from the MinION. The MinION can be monitored in real time and the sequencing stopped once sufficient data has been received, such as mitochondrial sequences for taxonomic identification, further reducing sequencing time. PCR based DNA barcoding is economical for taxonomic classification purposes of multiple species, however, genome skimming using the MinION sequencer provides genomic information for a single species which will facilitate taxonomic classification, determination of population structure and size as well as accurate phylogenetic assessment. In addition, MinION sequencing allows researchers to maintain custody of samples during the entire process, whereas Sanger sequencing may require the samples to be de-identified before being sent to a sequencing facility for high-stakes studies.

The ability to perform sequencing experiments, assessing multiple genomic regions and determining taxonomic identities, irrespective of species or sample type, in remote locations provides a significant advantage for wildlife research and could make MinION a preferred choice for sequencing dependent upon the application of sequence data. Future studies in our laboratory will determine whether first, additional chondrichthyan and wildlife taxa can be analyzed in a fashion similar to *C*. *falciformis* using methods described here on the MinION sequencer and second, whether multiple specimens can be analyzed using our methods on a single MinION flowcell by barcoding each specimen, to reduce cost and increase efficiency. However, when sequencing multiple specimens simultaneously, reduced sequencing coverage of large chondrichthyan genomes could limit analyses of multiple samples with the MinION.

Sequencing large genomic sections with long read capabilities of the MinION will increase the size and diversity of sequence databases and improve annotation of sparsely annotated loci through gene mapping, and annotation. This will improve application of current and future genomic data for taxonomic, phylogenetic, metabolic or population studies. For example, currently the COI and NADH2 barcodes are well curated within the Genbank^41^ and BOLD^42^ databases for chondrichthyan species, but availability is sparse for other mitochondrial loci such as the 12s and 16s rRNA genes and many nuclear loci including ITS2. Therefore, taxonomic identification, phylogenetic and population studies in Chondrichthyes often rely on COI and NADH2 assessments, limiting the accuracy and depth of these analyses. Increased ease of Genome skimming or whole genome sequencing, using our methods will make larger regions of chondrichthyan genomes accessible and will help populate genetic databases. This approach will increase accuracy of phylogenetic, population and kinship assessments and will significantly reduce data deficiencies in Chondrichthyes for conservation and management purposes.

### Analyses

We compared efficiency and expediency of two different workflows **(**Fig. 2**)**, one with a GUI (Workflow I) and the other command line based (Workflow II), for sequence analyses of MinION data. Mitochondrial sequences obtained from both workflows were 98% identical, differing by 400 bp in length. Phylogenetic analyses with both sequences resulted in an identical taxonomic classification of the unknown sample. Minor differences in mitochondrial sequences could be attributed to differences in the mapping and assembly algorithms underlying the assembly tools used in the two workflows. Workflow II also used more rigorous quality controls and trimmed sequences before assembly which may have led to the loss of some sequence base pairs.

The MinION Workflow I we developed allowed quick access to mitochondrial genomes, while Workflow II allowed access to nuclear genetic loci, phylogenetic analyses of which corroborated results from the mitochondrial analyses and allowed further investigation into phylogeny of the genus. Since mitochondrial genomes from Workflow I and II were comparable for taxonomic identification, we recommend using Workflow 1 **(**Fig. 2) **for field studies with an urgent timeline**. Here a database of chondrichthyan sequences from BOLD and Genbank is first curated in Geneious for offline access. DNA sequences from unknown specimens are obtained from the MinION and directly mapped to reference mitochondrial sequences using Geneious software. The mapped mitochondrial sequences are then aligned with reference sequences in the offline databases for taxonomic identification making it as streamlined as upload and align sequence to identify species. The use of multiple mitochondrial barcodes ensures the highest level of accuracy. These analyses are straightforward and do not require internet connectivity, or extensive bioinformatics capabilities or computing power. Workflow I can be used to train researchers in accurate taxonomic identification of species without using computer programing.

Workflow II can be used in advanced stages of analyses not limited by computing power, bioinformatics expertise and time. The nuclear and mitochondrial sequences obtained from Workflow II can be used to corroborate results obtained using just mitochondrial makers in Workflow I and to obtain finer phylogenetic resolution among species. Population genetic studies may also be undertaken at this stage.

### Phylogenetics

Our phylogenetic analyses using mitochondrial genomes and nuclear loci of the genus *Carcharhinus* consistently support placement of the unknown sample as sister to *C*. *falciformis* **(**Fig. 5A,B**)** and demonstrate the utility of genome skimming techniques for taxonomic identification of unknown samples using multiple genetic loci. Additionally, analyses of mitochondrial and nuclear data are congruent with previous work^9,43^ and strongly support sister species relationships of sampled taxa throughout the genus **(**Fig. 6**)**. Albeit our analyses allow assessment of sister species relationship with denser sampling of taxa throughout the Carcharhinus genus.

The inclusion of multiple mitochondrial genes and nuclear loci provided higher statistical support and further evidence that *P*. *glauca* is nested well within Carcharhinus, advancing previous work in the genus that used fewer markers^9,13^ and subsequently had weaker statistical support^43^. Our nuclear and mitochondrial data support a sister relationship between *P*. *glauca* and *C*. *amblyrhynchos* with *C*. *falciformis* as outgroup. These data exemplify the statistical power achieved using diverse genetic and intergenic markers which were accessible in the current study from using a genome-wide sequencing approach.

Deeper nodes relating four taxa-*C*. *falciformis*, *P*. *glauca*, *C*. *amblyrhynchos* and *C*. *wheeleri* are not well supported in previous studies^9^ or in our analyses (<0.95 PP), because of mito-nuclear discordance, missing data, and lack of coverage of mitochondrial loci. As Bayesian phylogenetic estimation techniques are often robust to missing data in a concatenated alignment^44^, we think that a substantial amount of missing data, as seen with Chondrichthyes, has led to poor support for these phylogenetic analyses. This once again, highlights the need for further genomic analysis. In addition, *Carcharhinus* spp. are known to contain cryptic species^45,46^ and thus increasing the genomic databases for these species to include regions such as Ultra Conservative Elements (UCE)^47^ will be beneficial to understanding the genetic diversity of the genus. In our concatenated analysis, the unknown sample was consistently represented on a very long branch **(**Fig. 6**)**. This stems from difficulties in aligning the long segment of the unknown sample ITS2 sequence obtained from the MinION to the ITS2 sequences available on GenBank which were much shorter and thus had limited species’ information. In the final concatenated alignment, the unknown sample ITS2 sequence showed numerous insertions not present in other samples. These insertions in the MinION derived sequences are potential evidence of sequence information that is missing in the shorter PCR derived Genbank sequences which may be restricted by polymerase processing rates, amplicon length restrictions or PCR artifacts resulting from repeat regions of template sequences such as the ITS2 region. MinION derived sequences are contiguous long reads of actual genomic DNA which does not go through an amplification cycle but is simply denatured and sequenced, and hence the unknown sample ITS2 sequences is not affected by amplification biases or restrictions of amplicon size, thus providing a higher quality analysis.

### *Carcharhinus falciformis*

*Carcharhinus falciformis* or silky shark is a pelagic species found in tropical waters in the Indian, Pacific, and Atlantic oceans^48^. This species is highly migratory, and has low fecundity, placing it at risk from incidental take in high seas fisheries. Threats to the species are exacerbated by demand for meat and fins in the international market. Despite some regional prohibitions, silky shark mortality is under-reported and largely unmanaged. Inclusion of the silky shark in CITES Appendix II^49^ is likely to bolster compliance with existing protections, complement existing commitments under the Convention on Migratory Species (CMS) and facilitate international cooperation toward more comprehensive international conservation measures, thereby enhancing the chances for sustainable use. However, CITES listing and other measures are ineffective if the trade in this species cannot be detected in international fin exports^4,50^. Methods and results presented here increase our ability to study silky sharks and increase traceability of this species in the fin trade.

A single complete mitochondrial genome sequence of 17,639 bp for *Carcharhinus falciformis* has been published^27^. A suspicious insertion of ~939 bp is evident when the sequence^27^ is compared to all *Carcharinus* spp. mitogenomes, which are at ~16,700 bp. The mitochondrial sequence for *Carcharhinus falciformis* presented in the current report **(**Fig. 4, Genbank accession #MK092088) provides the first complete and accurate mitochondrial sequence for the species. Further, the investigation into the nuclear and mitochondrial sequences presented in the current study provided better phylogenetic resolution for the *Carcharhinus* genus. The immunoglobulin and endocrine function encoding nuclear sequences will provide an understanding of evolutionary relationships, immune function development and physiology of the species in future studies.

## Conclusions

We have bridged substantial knowledge gaps in chondrichthyan genetics through work presented in this report. Use of the MinION in the field is a potential game-changer because it can obtain vast amounts of data in areas with high data deficiency and high fishing effort with respect to Chondrichthyes. It can facilitate accurate identification and ecological investigation of existing as well as new and rare species, without the need for *a priori’* sequence information. These methods will improve the accuracy of biodiversity estimates in areas with minimal infrastructure, thus addressing the data deficiency and knowledge gaps affecting Chondrichthyes where data is most needed. Sequencing of larger regions of chondrichthyan genomes as opposed to single barcode sequencing will allow wider ecological investigations for all Chondrichthyes such as population genetic studies, stock assessments, biogeographic and kinship studies, thus increasing the depth of our understanding of chondrichthyan populations. The whole genome provides insights into shark metabolism and health status. Data regarding population structure within geographically isolated populations of the same species can allow assessment of population stocks and inform conservation and management of the species. Last, the genome sequencing and analyses methods presented in the current report are independent of PCR bias, and have sequencing capacity across a wide GC %, making them amenable to ecological investigations on a variety of non-model species. The workflows presented here, can be used effectively to build capacity from molecular ecology research among wildlife researchers and fisheries scientists in a variety of infrastructure settings.

## Methods

### Sampling and DNA extraction

Shark samples (n = 30) consisting of fin tissue ~5 mm^3^ in size were collected from dismembered shark specimen at a fish market in Veraval, Gujarat, India in April 2017 (Fig. 1). Samples were stored in DNA/RNA Guard^TM^ Zymoresearch at ambient temperature during collection and then stored at −20 °C until processing. DNA extraction was conducted using Quick-DNA^TM^ Plus extraction kit (Zymoresearch^TM^) with an extended 3-hour proteinase K digestion at 55 °C. Of these 30 samples, one was prioritized for analyses with the MinION sequencer and the rest were stored for analyses at a later time.

### Morphological identification

Photo identification of the specimen was performed using protocols described by Ebert *et al*.^51^ and based on intact features of the dismembered specimen, including head, gills, and fins.

### PCR Amplification and Analyses

PCR amplification of three gene fragments from the Cytochrome Oxidase 1 and NADH2 genetic loci was performed to resolve the taxonomic identity of the specimen at the species level using protocols described previously. PCR amplification of a 633 bp and 160 bp fragment of the COI regions was performed using methods described by Ward *et al*.^6^ and Fields *et al*.^8^, and that of a 1044 bp NADH2 fragment was done using protocols described by Naylor *et al*.^9^. PCR amplification was followed by PCR clean up with Zymoresearch^TM^ DNA Clean and Concentrator Kit and uni-directional sequencing using BigDye Terminator chemistry on an ABI 3730xl genetic analyzer (Applied Biosystems, Life Technologies). The resulting sequence was used to determine the taxonomic identity of the chondrichthyan specimen using the Barcode of Life Database (BOLD)^26^.

### Library preparation and Sequencing on the MinION

In order to access the entire length of multiple nuclear and mitochondrial genomes in a PCR-independent fashion, genome skimming using the MinION device was performed. The extracted DNA was measured using a Qubit^TM^ spectrophotometer. Genomic DNA (~400 ng) was used directly for library preparation with the Rapid Sequencing kit (SQK-RAD003) from ONT using the manufacturer’s protocols. The library preparation and sample loading took 15–30 minutes in total and required access to a thermocycler or hot water bath incubator, pipettes and appropriate tips (1000 μl, 200 μl, and 10 μl), and molecular grade water. Bound DNA was loaded directly into the sample loading port of the FLO-MIN106 (R9.4 SpotON) flowcells after priming of the flowcells. Sequencing was continued for 36 hours, however, sequences acquired were monitored throughout the 36  hours and mitochondrial barcodes could be obtained starting at 8 hours from start of sequencing.

### Bioinformatics Analyses

Sequence data were analyzed using two parallel workflows (Fig. 2). For the first workflow, FASTQ files were uploaded into Geneious^®^ software version 11.1.2^31^. Mitochondrial sequences were mapped directly to reference chondrichthyan mitochondrial sequences from Genbank^®^ using Geneious Read mapper^52^ and the resulting contig was used to obtain a contiguous mitochondrial sequence for the sample analyzed.

For the second workflow, reads were assembled using CANU (Version 1.7)^53^ with flags that set the genome size as 16 kb, trimmed the minimum read length to 500 bp, and set the reads as coming from the MinION. Contigs were aligned using BLAST^54^ against the nt nucleotide database to identify mitochondrial and nuclear sequences. Using CANU reads from a circular genome assemble into a linear contig larger than the original genome with repeated sequences at both ends. Therefore, the mitochondrial genome was circularized using Samtools (version 1.8)^55^ and Nucmer (version 4.0.0 beta2)^56^ was used to identify repeated sequences in the mitochondrial contigs. Gepard (version 1.4)^57^ was used to produce dotplots and verify the circularization. This workflow is available in our associated Jupyter notebook: https://github.com/Adrian-Cantu/git-presentations/blob/master/shark_mito/Mito_mapping.ipynb.

Mitochondrial contigs from both workflows were annotated using Mitos^28^, a web-based server for *de novo* annotation of mitochondrial genomes, and Mito-annotator (Mitofish database)^29^, a tool for annotation of fish mitochondrial genomes. tRNA identification was conducted by ARWEN — a program to detect tRNAs in metazoan mitochondrial genomes^30^. CGView was used to determine GC rich regions and GC skew^58^.

### Taxonomic identification and Phylogenetics

We investigated the taxonomic identity of our sample using whole mitochondrial genomes and nuclear DNA (ITS2) comparisons to previously sequenced *Carcharhinus* from the NCBI database^41^. We reconstructed phylogenetic relationships within the genus *Carcharhinus* in parallel, first using complete mitochondrial genomes when available (n = 14), and second, using concatenated sequences for three protein-coding mtDNA genes (COX1, NADH2, and ND4), and one nDNA locus (ITS2), (n = 33 species within *Carcharhinus*). *Galeocerdo cuvier* and *Prionace glauca* were used as the outgroup in the two phylogenetic analyses. Sequences were acquired from GenBank **(**Supplementary Tables S1, S2 and S3**)** and aligned using MUSCLE 3.8.31^59^. The mitochondrial alignments were done in two different ways, using the 13 protein-coding regions of the mitochondrial genome or the complete mitogenome including coding and non-coding regions. Alignments were partitioned by gene and codon position. We used PartitionFinder 2.1.1^60^ on XSEDE tool on CIPRES^61^ to identify the appropriate partitioning schemes and models of substitution using the greedy search algorithm. Maximum-likelihood phylogenies were estimated in RAxML-HPC v8.2.10^62^ on XSEDE run on CIPRES Scientific Gateway using a separate GTR + Γ model specified for all partitions. We ran 10 searches beginning with different random stepwise addition parsimony trees with the default RAxML algorithm. Support values for the best tree were estimated using non-parametric bootstrapping implemented using the auto-MRE stopping criterion. We estimated Bayesian phylogenies in MrBayes v3.2.6^63,64^, on XSEDE run on CIPRES Scientific Gateway using the partitioning scheme specified by PartitionFinder2. We used unlinked GTR substitution model across partitions and accommodated rate heterogeneity among sites by using a Γ distribution. We assessed convergence in Tracer v1.6.

## Supplementary information

Supplementary Dataset1

Supplementary Table S1

Supplementary Table S2

Supplementary Table S3

Supplementary Table S4

## Data Availability

All data generated or analyzed during this study are included in this published article (and its Supplementary Information files).
