# Supplementary material for: ‘Genome skimming’ with the MinION hand-held sequencer identifies CITES-listed shark species in India’s exports market
Source: Sci Rep. 2019 Mar 14;9:4476. doi: 10.1038/s41598-019-40940-9 (PMC6418218; doi:10.1038/s41598-019-40940-9)
Supplement: Supplementary file 1 — Supplementary Dataset1 [file 41598_2019_40940_MOESM1_ESM.pdf]

Supplementary Figures
Manuscript Title:

'Genome skimming' with the MinION hand-held sequencer identifies CITES-listed shark species in India's exports market.

Authors:
Shaili Johri (First author)[1]
Jitesh Solanki[2]
Vito Adrian Cantu[3]
Sam Fellows[1]
Robert Edwards[3]
Isabel Moreno[1]
Asit Vyas[2]
Elizabeth Dinsdale (Corresponding author)*

Author information:
1. Department of Biology
   5500 Campanile Dr.
   San Diego State University
   San Diego, CA 92128

2. College of Fisheries Science
   Rajendra Bhuvan Road,
   Junagadh Agricultural University
   Veraval, Gujarat 362266, India.

3. Computational Sciences Research Center
   5500 Campanile Drive
   San Diego State University
   San Diego, CA 92128

***Corresponding author information:**
Elizabeth A. Dinsdale
Department of Biology
San Diego State University
5500 Campanile Dr.
San Diego, CA 92128
Tel: 619-594-5623,
Email: edinsdale@mail.sdsu.edu

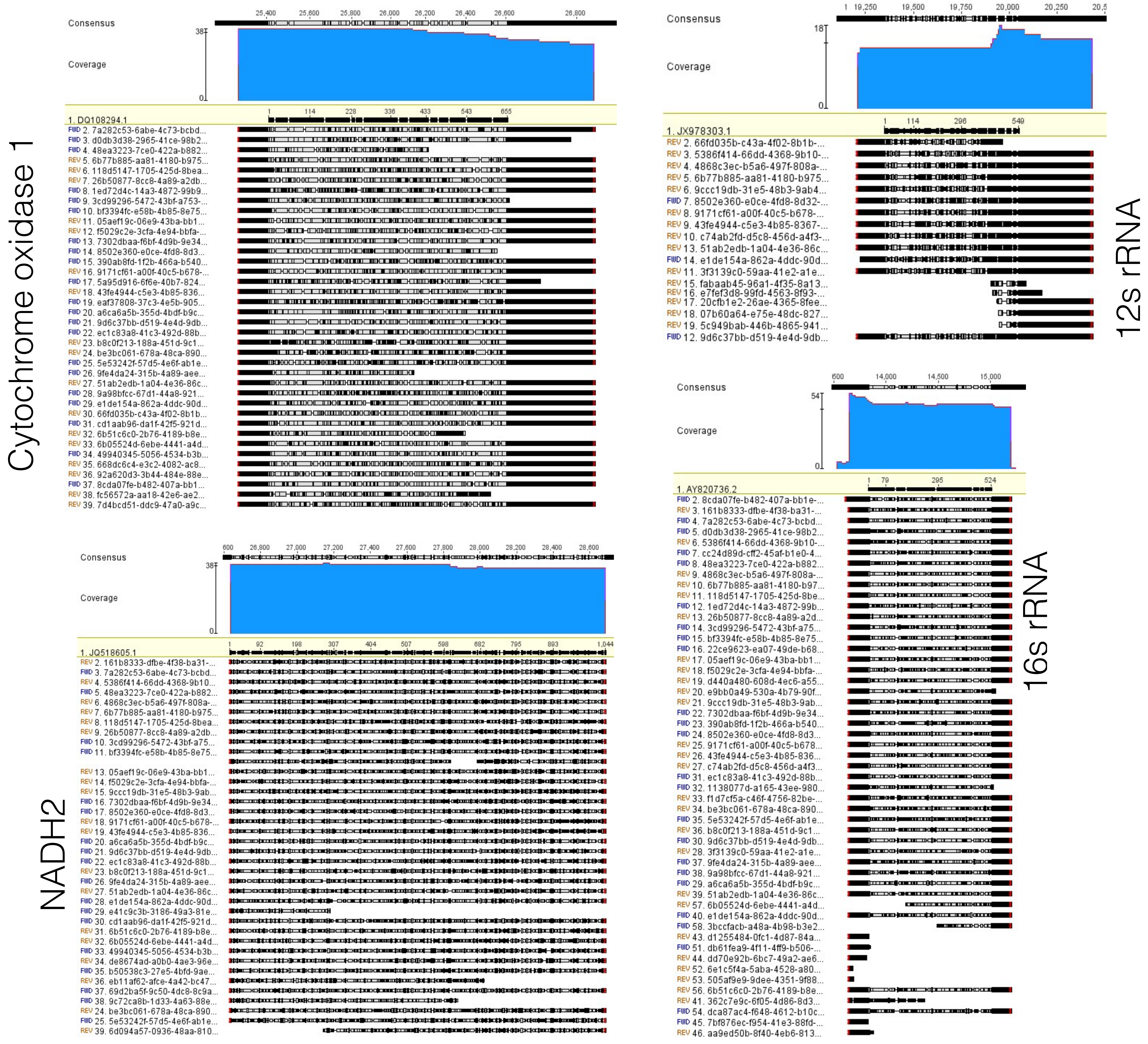

Supplementary Figure S1A: Sequencing coverage for mitochondrial barcodes.
regularly used individual mitochondrial barcodes Cytochrome oxidase 1 (35x), NADH2 (35x), 12s (15x) and 16s rRNA (40x) obtained from workflow 1.

Contig	Nuclear Gene	Coverage
tig00000001	internal transcribed spacer 2, and 28S ribosomal RNA gene, partial sequence	3x
tig00000003	HOXD sequence	> 60x
tig00000010	mitochondrion, complete genome	19.4x
tig00000017	antithrombin (serpinc1) gene, complete cds	4x
tig00000019	Ig lambda light chain gene, complete cds	>10x
tig00000756	HOXB sequence	1x
tig00000758	IL-1 gene for interleukin-1beta, complete cds	1x
tig00000761	recombination activating gene 1 (RAG1) gene, partial cds; and recombination activating gene 2 (RAG2) gene, complete cds	>100x
tig00000762	recombination activating gene 1 (RAG1) gene, partial cds; and recombination activating gene 2 (RAG2) gene, complete cds	1x

Supplementary Figure S1B: Sequencing coverage for nuclear and mitochondrial sequences obtained with Workflow II.

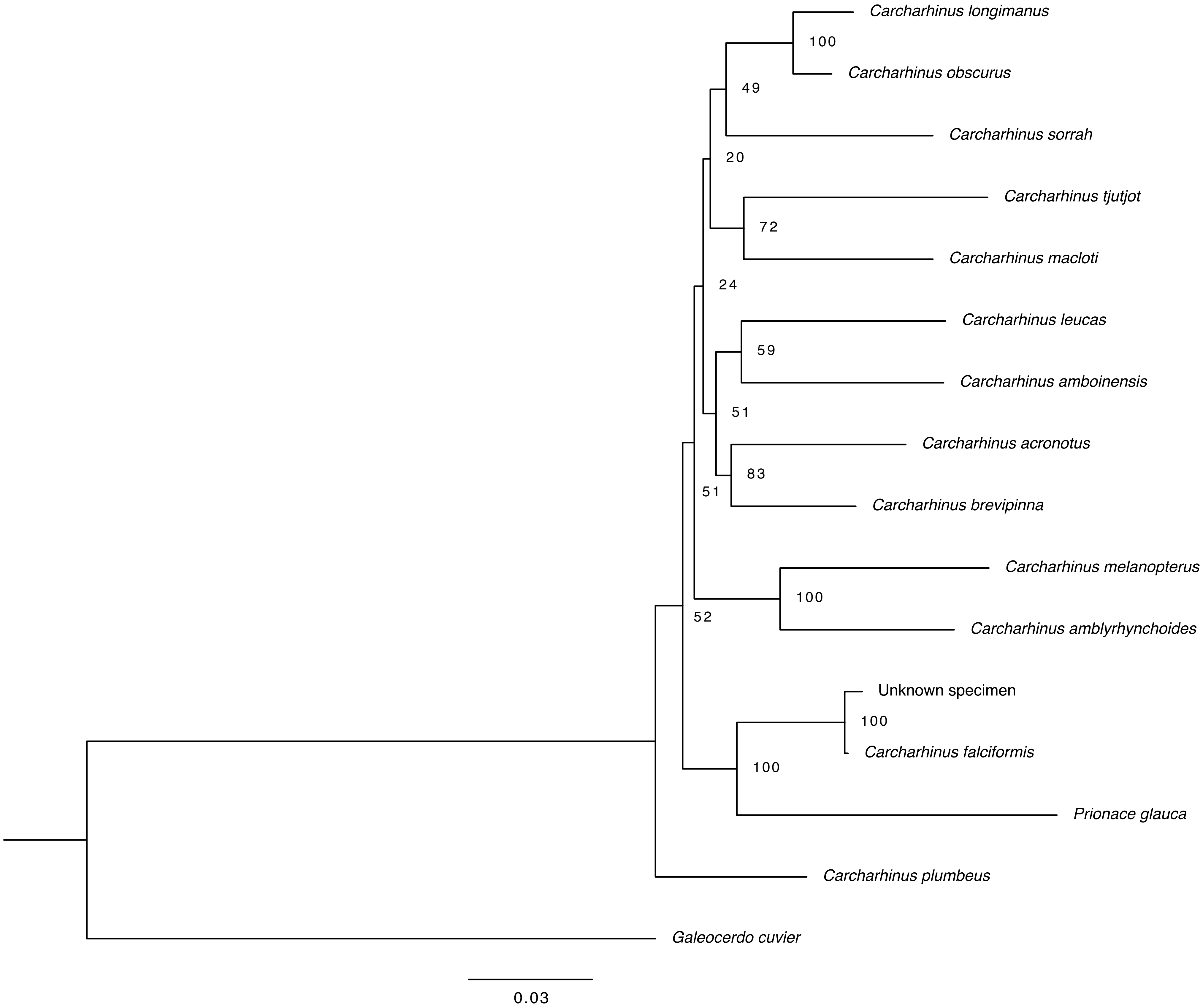

Supplementary Figure S2A: Maximum-likelihood estimate of relationships among Carcharhinus spp. using complete mitogenomes.
Maximum-likelihood phylogenetic estimate from mitochondrial genome from 13 *Carcharhinus* species and, *Prionace glauca*, and *Galeocerdo cuvier* as an outgroup. The unknown sample clusters with *C. falciformis.* Numbers at nodes are bootstrap support values.

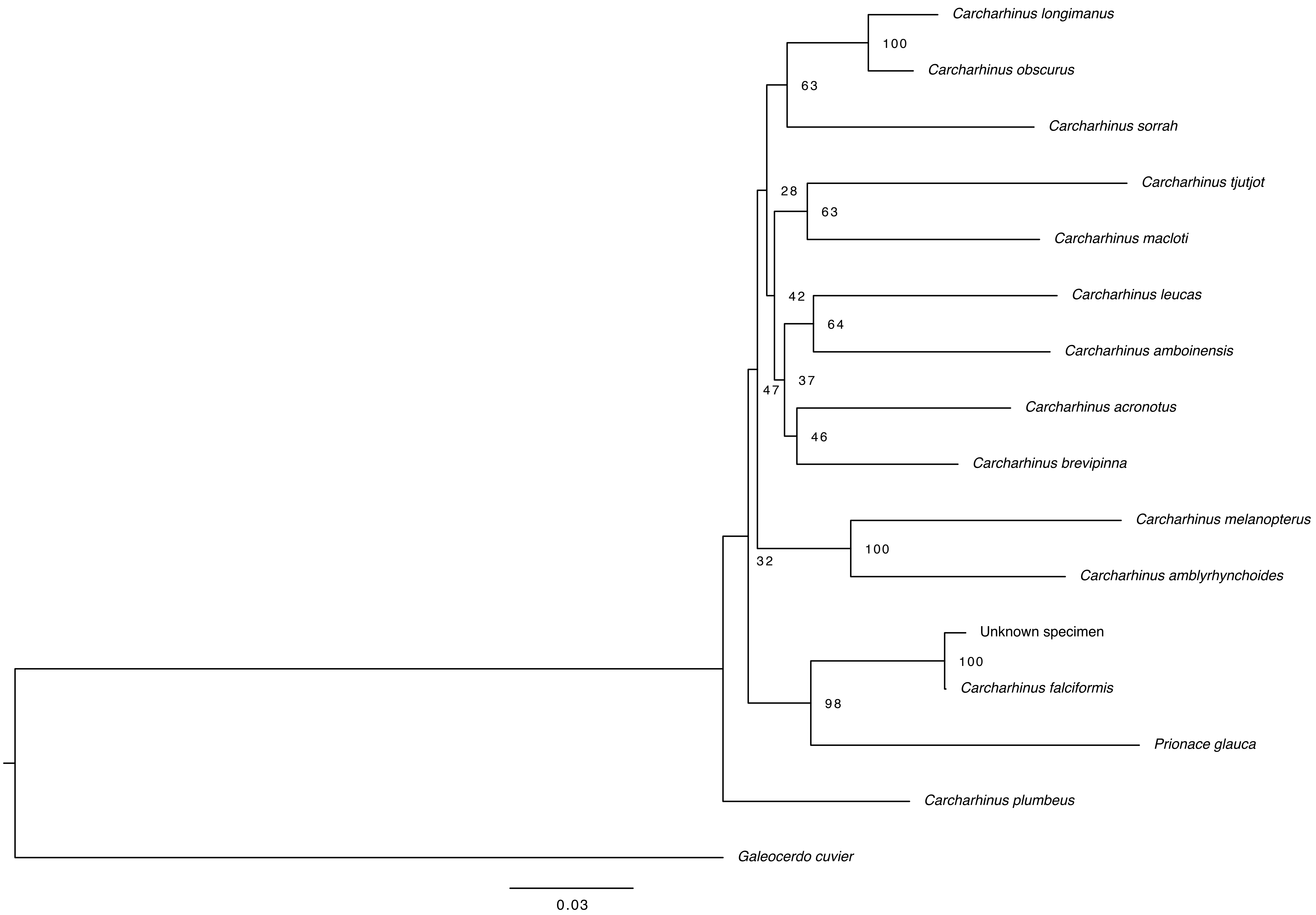

Supplementary Figure S2B: Maximum-likelihood estimate of relationships among Carcharhinus spp. using mitochondrial protein coding genes. Maximum-likelihood phylogenetic estimate from 13 protein-coding mitochondrial genes from 13 *Carcharhinus* species and, *Prionace glauca*, and *Galeocerdo cuvier* as an outgroup. The unknown sample clusters with *C. falciformis.* Numbers at nodes are bootstrap support values.

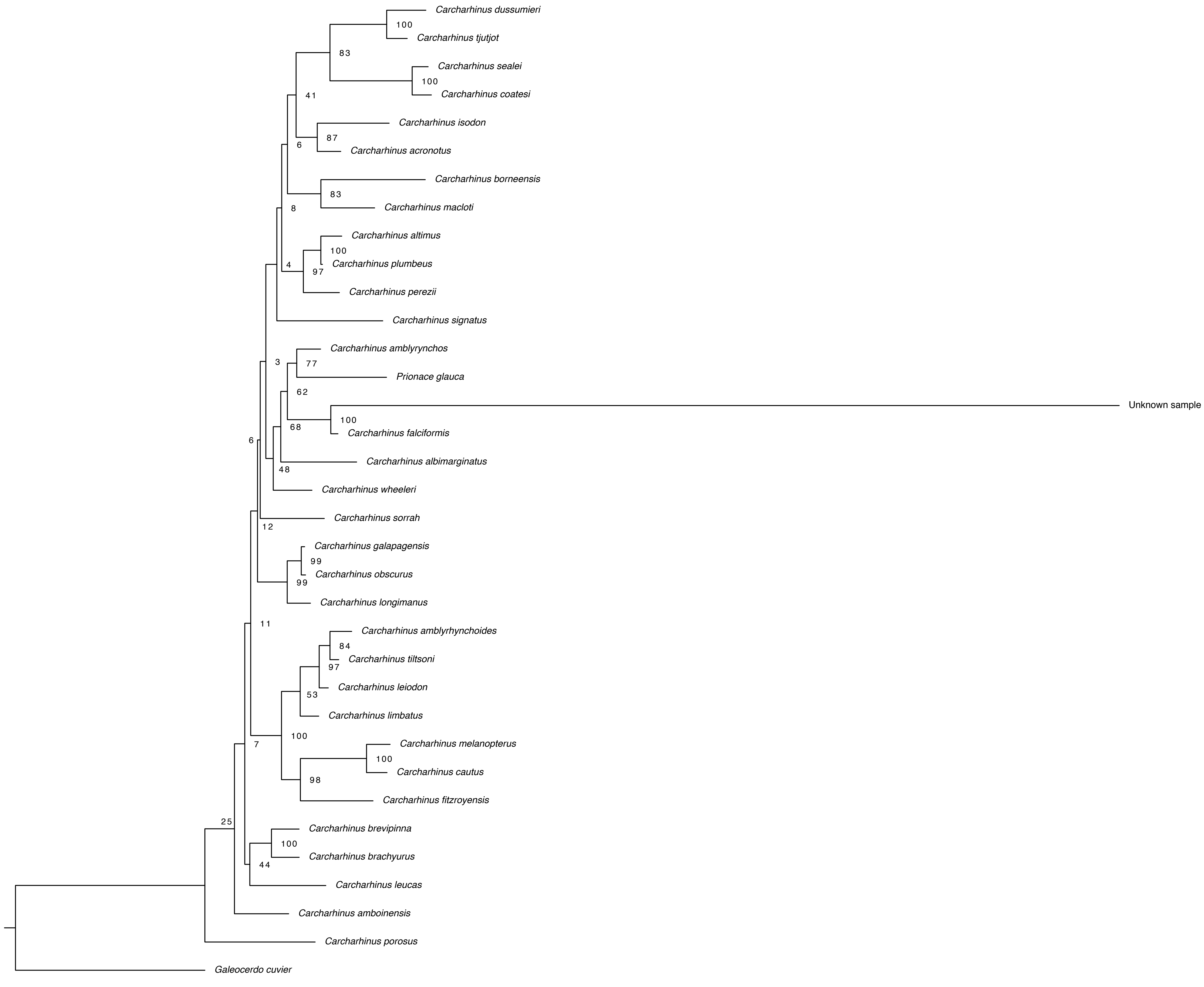

Supplementary Figure S2C: Maximum-likelihood estimate of relationships among Carcharhinus spp. using mitochondrial and nuclear genes. Maximum likelihood phylogenetic estimate from concatenated alignment of three mitochondrial protein coding loci (NADH2, COI, NADH4) and one nuclear locus (ITS2) from 33 Carcharhinus species and, *Prionace glauca*, and *Galeocerdo cuvier* as an outgroup. The unknown sample clusters with *C. falciformis*. Numbers at nodes are bootstrap support values.
